# The efficacy and safety of high-flow nasal cannula therapy in patients with COPD and type II respiratory failure: a meta-analysis and systematic review

**DOI:** 10.1186/s40001-021-00587-7

**Published:** 2021-10-14

**Authors:** Zhiping Xu, Lingxia Zhu, Jingye Zhan, Lijun Liu

**Affiliations:** Department of Emergency and Critical Care Medicine, The Second Affiliated Hospital of Soochow University, No.1055, San Xiang Road, Suzhou, Jiangsu Province China

**Keywords:** HFNC, COPD, Respiratory failure, Ventilation, Treatment, Review

## Abstract

**Background:**

High-flow nasal cannula (HFNC) and noninvasive ventilation (NIV) have been used for the treatment of COPD and respiratory failure in clinical settings. We aimed to evaluate the efficacy and safety of HFNC therapy in patients with COPD and type II respiratory failure, to provide evidence to the clinical COPD management.

**Methods:**

We searched Cochrane et al. databases up to Dec 31, 2020 for randomized controlled trials (RCTs) on the use of HFNC therapy in patients with COPD and type II respiratory failure. Two researchers independently screened the literature according to the inclusion and exclusion criteria, and evaluated the quality of the literature and extracted data. We used Revman5.3 software for statistical analysis of collected data.

**Results:**

A total of 6 RCTs involving 525 COPD and type II respiratory failure patients. Meta-analyses indicated that compared with NIV, HFNC could significantly reduce PaCO_2_ level (MD = − 2.64, 95% CI (− 3.12 to − 2.15)), length of hospital stay ((MD = – 1.19, 95 CI (− 2.23 to − 0.05)), the incidence of nasal facial skin breakdown ((OR = 0.11, 95% CI (0.03–0.41)). And there were no significant differences between the two groups in PaO_2_ ((MD = 2.92, 95% CI (− 0.05 to 5.90)), incidence of tracheal intubation ((OR = 0.74, 95% CI (0.34–1.59)) and mortality (OR = 0.77, 95% CI (0.28–2.11)).

**Conclusions:**

HFNC is more advantageous over NIV in the treatment of COPD and type II respiratory failure. Future studies with larger sample size and strict design are needed to further elucidate the role of HFNC in COPD and respiratory failure.

## Background

Chronic obstructive pulmonary disease (COPD) is a common chronic disease characterized by persistent airflow limitation [1]. There are nearly 100 million COPD patients in China, and the prevalence of COPD in people over 40 years old is 13.7% [2, 3]. Acute exacerbation of COPD and complications are the main causes of death [4]. Oxygen therapy is the main treatment method for patients with COPD and hypoxemia [5]. Traditional oxygen therapy uses low-flow oxygen, but some patients' hypoxia or hypercapnia are difficult to correct, and noninvasive ventilation (NIV) has gradually become the gold standard for the treatment of patients with AECOPD and type II respiratory failure [6]. However, the design of NIV masks and nasal masks has disadvantages such as facial compression, affecting patient communication, eating and sleep [7, 8]. The poor comfort and mask intolerance can easily lead to tracheal intubation and cause NIV treatment failure [9].

High-flow nasal cannula (HFNC) is a new type of non-invasive respiratory assistance method. It can reduce PaCO_2_ by using the flushing effect of high flow and adjust the oxygen flow and oxygen concentration separately to avoid the high concentration of oxygen causing respiratory depression in patients [10, 11]. HFNC can improve ventilation and oxygenation and improve comfort by providing oxygen that is heated and humidified with precise oxygen concentration [12]. Studies [13–15] have shown that the use of HFNC oxygen therapy for COPD patients can reduce the frequency of exacerbations and improve exercise capacity and quality of life. Some studies [14, 16] have also shown that HFNC can improve oxygenation in patients with COPD. However, several studies have shown that HFNC can be used in patients with AECOPD and type II respiratory failure, but it has no effect on the incidence of patients’ tracheal intubation, length of hospital stay, and incidence of adverse events. The effects and safety of HFNC treatment in patients with COPD and type II respiratory failure remain unclear. Therefore, we aimed to conduct a meta-analysis of the application of HFNC in the treatment of patients with COPD and type II respiratory failure, to evaluate the efficacy and safety of HFNC, thereby providing a basis for the clinical treatment of COPD and respiratory failure.

## Methods

We conducted and reported this meta-analysis and systematic review in compliance with the preferred reporting items for systematic reviews and meta-analyses (PRISMA) [17].

### Search strategy

We searched the Cochrane Clinical Trials Database, EMBASE, CINAHL, PubMed, web of science, Wanfang, and Weipu Knowledge Network for randomized controlled trials(RCTs). The search time limit is from the establishment of the database to Dec 31, 2020. We used following search terms: (“high flow nasal cannula” OR “HFNC” OR “high flow nasal oxygen” OR “high flow oxygen therapy” OR “high flow nasal cannula oxygen therapy”) AND (“Pulmonary Disease” OR “Chronic Obstructive” OR “Chronic Obstructive Pulmonary Disease” OR “Chronic Obstructive Lung Disease” OR “COPD” OR “Chronic Obstructive Airway Disease” OR “COAD” OR “chronic airflow obstructions” OR “pulmonary emphysema”). At the same time, we traced the relevant references of the included literature as a supplement to further search the possibly relevant literature.

### Inclusion and exclusion criteria

The RCT inclusion criteria for this meta-analysis were: ① the research populations were COPD patients with type II respiratory failure; ② age ≥ 18 years; ③ intervention measures: HFNC was used in the experimental group, and NIV was used in the control group; ④ the main outcome indicators such as PaCO_2_, PaO_2_ et al. had been reported; ⑤ RCT study design; ⑥ Chinese and English literature.

The exclusion criteria were: ① randomized crossover studies; ② before-and-after control study; ③ studies with incomplete data, which could not be extracted and included for synthesized analysis.

### Literature screening

We imported the retrieved literatures into the ENDNOTE software, and we checked the duplicates based on the title, authors and year of the documents. Two researchers independently reviewed each of the retrieved documents. According to the inclusion and exclusion criteria, the documents were initially screened by reading titles and abstracts, and then the preliminary screening documents were further screened by reading the full text. If any differences were encountered, two authors discussed for consents. If the negotiation still could not be unanimous, and a third researcher would resolve by arbitration.

### Quality evaluation

The quality of RCT was evaluated using Cochrane’s risk of bias assessment tool [18], including evaluation of random sequence generation, allocation concealment, blinding of patients and interveners, blinding of outcome measurers, incomplete outcome data, selective reports and other potential bias. Each item was rated as “low risk”, “high risk” or “unclear”. When the evaluation results of the two investigators were inconsistent, the third investigator would make the decision.

### Data processing

We used Revman5.3 software for statistical analysis of data, and used *I*^2^ statistics and Cochrane *Q* test to assess whether there was heterogeneity between studies. The Cochrane *Q* test showed statistically significant heterogeneity when *P* < 0.1 and *I*^2^ ≥ 50%, and the random effects model was used. When the heterogeneity between the studies was not significant (*P* > 0.1, *I*^2^ < 50%), the fixed effects model was used. Binary data were expressed by odds ratio (OR) and 95% confidence interval (95% CI), and continuous data were expressed by weighted mean difference (MD). When *P* < 0.05, the difference between groups was statistically significant.

## Results

### Included RCTs

**Fig. 1:**
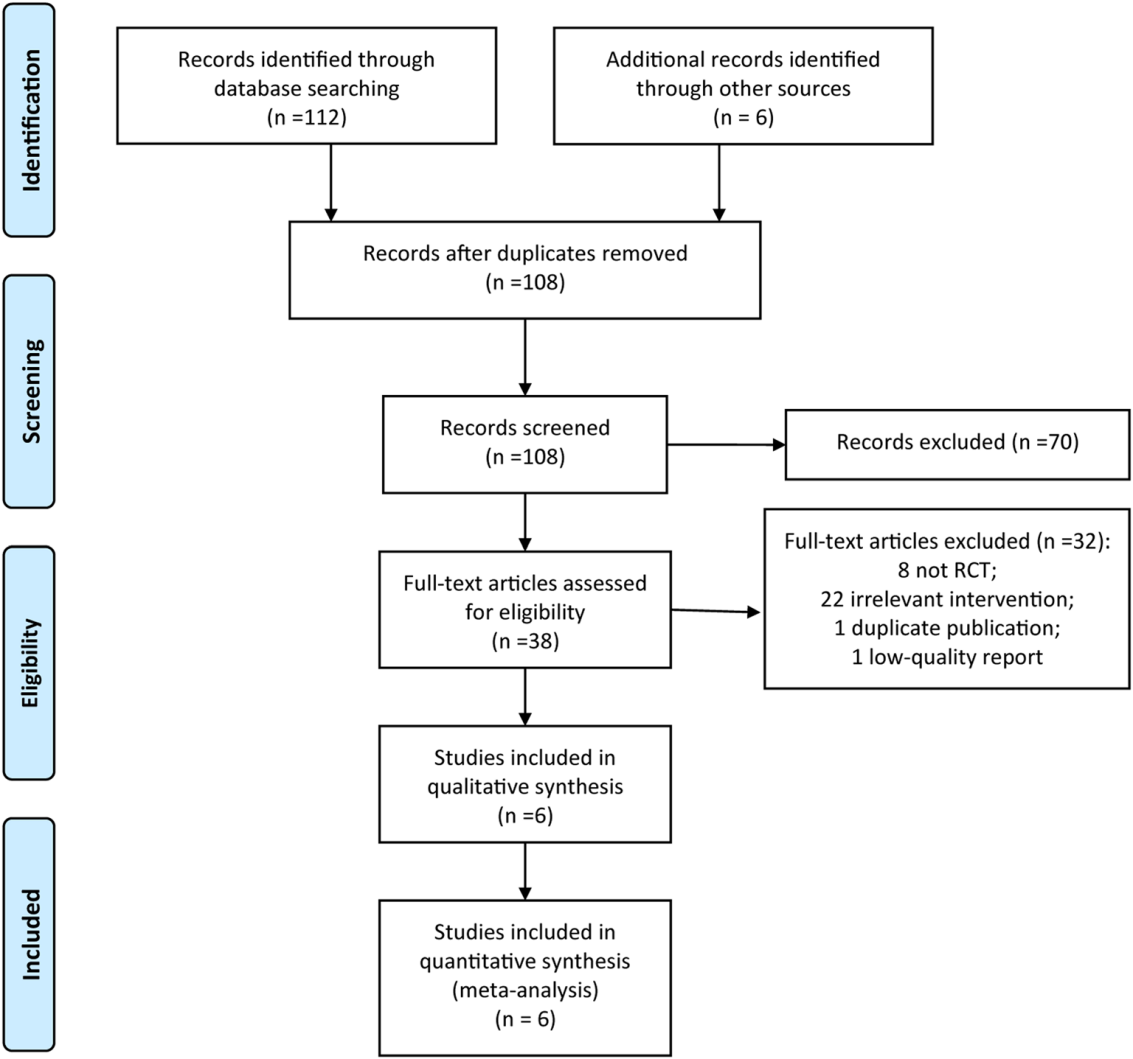
The PRISMA flow diagram of study selection

One hundred and eight related documents were initially retrieved through the databases after removing duplicate reports, after reading the titles and abstracts, 38 reports were sent out for full-text review. 32 studies were excluded since the documents that did not meet the inclusion criteria. Finally, 6 RCTs [19–24] were included. The study selection process is shown in Fig. 1. Of the 6 included RCTs, a total of 525 COPD patients were included, 259 patients underwent the HFNC treatment, and 266 patients underwent NIV treatment. The basic information of the included RCTs is shown in Table 1.

**Table 1:** The characteristics of included RCTs

RCT	Country	Sample size		Intervention		Outcomes
		Experimental group	Control group	Experimental group	Control group	
Ping 2018	China	47	46	HFNC	NIV	Incidence of tracheal intubation, mortality, incidence of nasal facial skin breakdown
Wang 2019	China	32	31	HFNC	NIV	Incidence of tracheal intubation, length of hospital stay
Jing 2018	China	38	37	HFNC	NIV	PaCO_2_, PaO_2,_ Incidence of tracheal intubation, length of hospital stay
Jiang 2019	China	50	50	HFNC	NIV	PaCO_2_, PaO_2,_ Incidence of tracheal intubation, length of hospital stay, mortality
Guo 2018	China	34	54	HFNC	NIV	PaCO_2_, PaO_2,_ Incidence of tracheal intubation, length of hospital stay
Chen 2020	China	48	48	HFNC	NIV	PaCO_2_, PaO_2_, pH, Incidence of tracheal intubation mortality

Arterial blood oxygen partial pressure (PaO_2_), carbon dioxide partial pressure (PaCO_2_)

### Quality of included RCTs

**Fig. 2:**
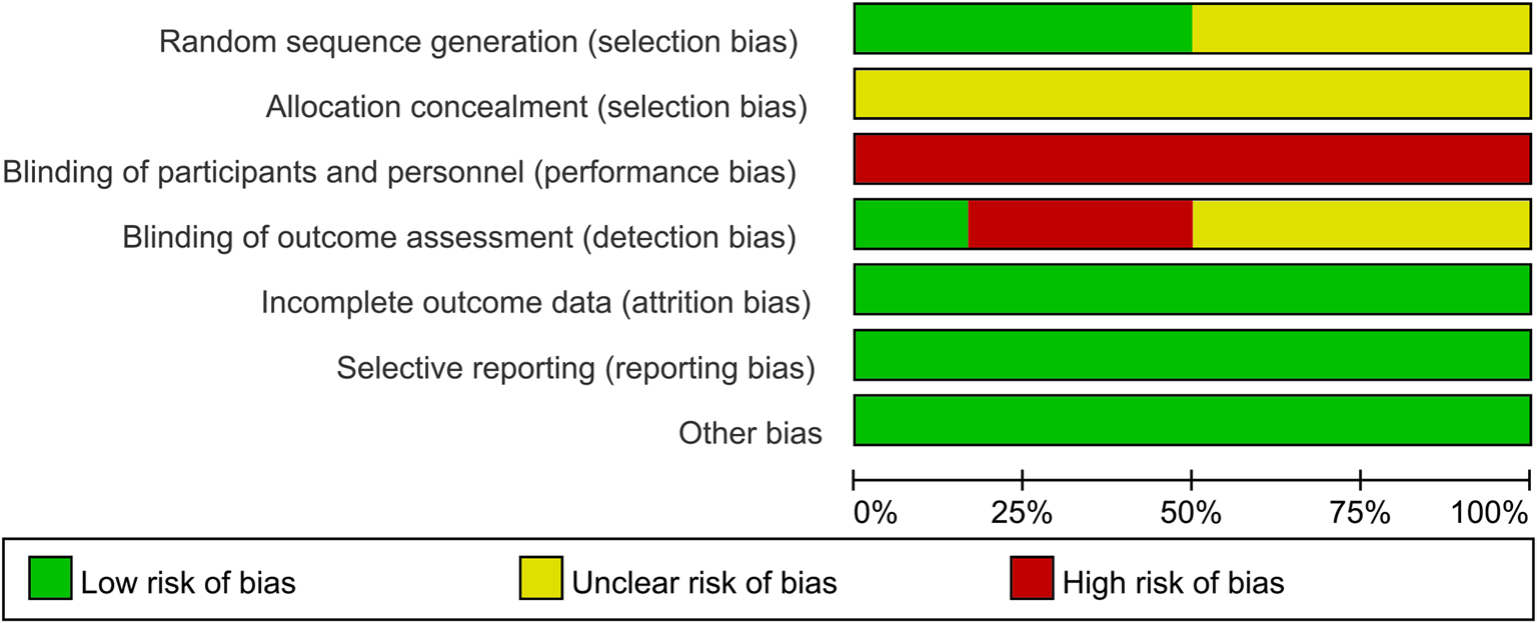
Risk of bias graph

**Fig. 3:**
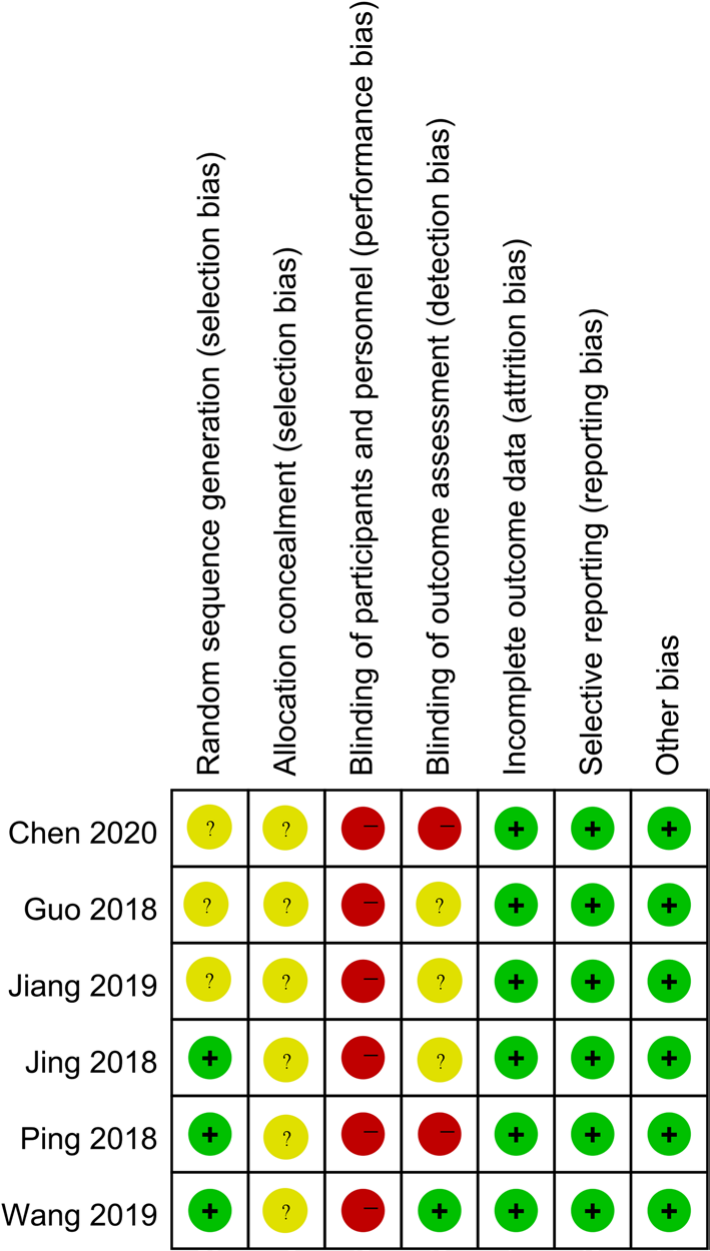
Risk of bias summary

Among the 6 included RCTs, 3 studies [19–21] clearly mentioned the method of using computer random sequence, and none of them mentioned allocation concealment. Due to the difference in oxygen therapy devices, it was difficult to blind the study patients and interveners, and all the research items were evaluated as having a high risk of bias. Since most of the outcome indicators are objective indicators, even if the evaluators are not blinded, they would not have a significant impact on the results. The blinding of outcome assessment in one study [19] was evaluated as low risk of bias. There were no other risks of bias in the results of all studies were found. The risk of bias results are shown in Figs. 2 and 3.

### Meta-analyses

#### PaCO_2_

**Fig. 4:**
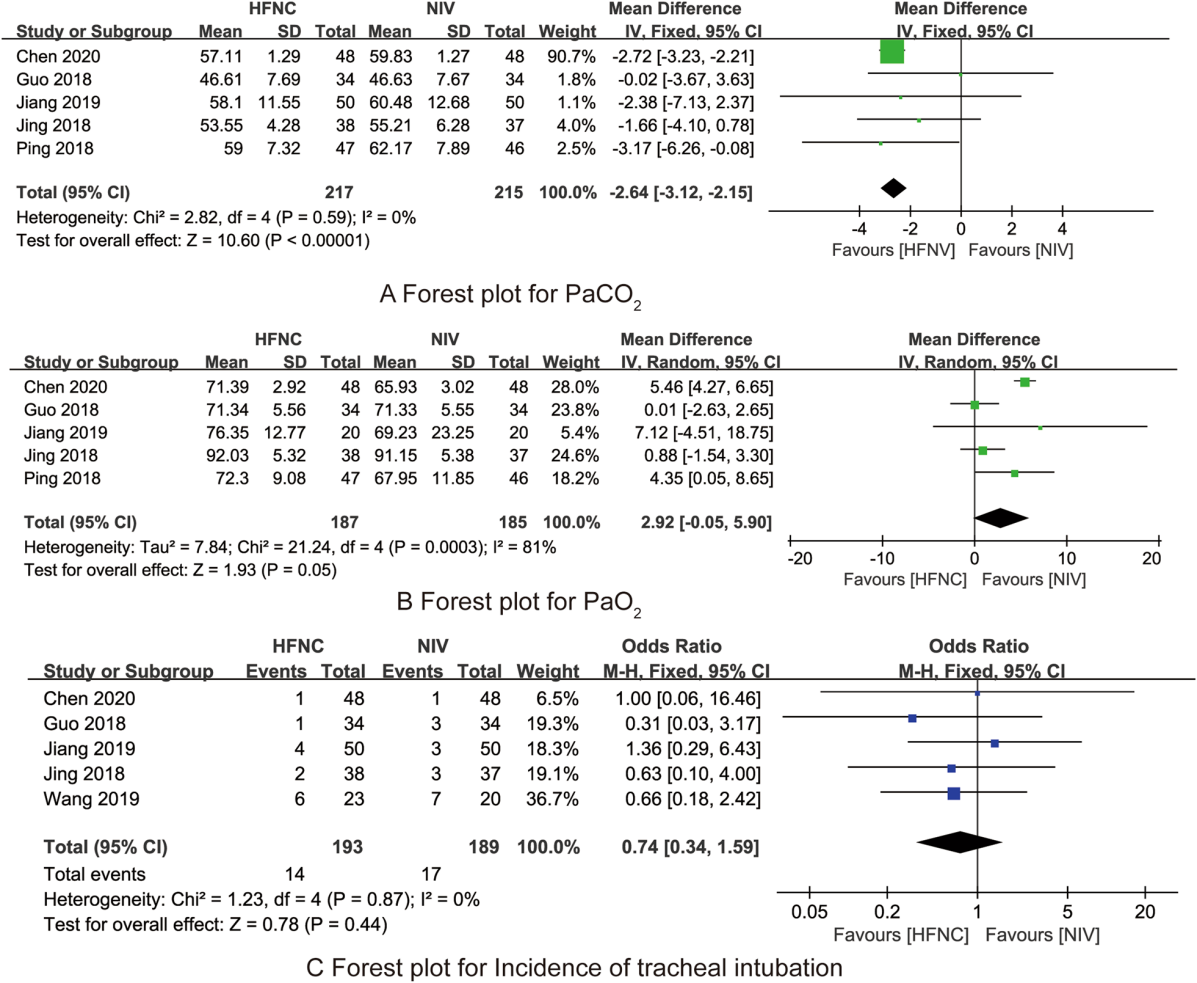
The forest plots for synthesized outcomes

Five RCTs [20–24] compared the effects of HFNC and NIV on PaCO_2_ after 24 h of oxygen therapy. There was no heterogeneity between the studies (*I*^2^ = 0%, *P* = 0.59), and the fixed effects model was used for analysis. The results showed that compared with NIV, HFNC could significantly reduce PaCO_2_ level (MD = – 2.64, 95% CI (– 3.12 to − 2.15), *Z* = 10.60, *P* < 0.001, Fig. 4A).

#### PaO_2_

Five RCTs [20–24] compared the effects of HFNC and NIV on PaO_2_ after 24 h of oxygen therapy. There was heterogeneity between the studies (*I*^2^ = 81%, *P* < 0.001), and the random effects model was used for analysis. The results showed that the difference between the two groups in PaO_2_ was not statistically significant (MD = 2.92, 95% CI (− 0.05 to 5.90), *Z* = 1.93, *P* = 0.05, Fig. 4B).

#### The incidence of tracheal intubation

Five RCTs [19, 20, 22–24] compared the effects of HFNC and NIV on the incidence of tracheal intubation. There was no heterogeneity between the studies (*I*^2^ = 0%, *P* = 0.87), and the fixed effects model was used for analysis. The results showed that the difference between the two groups in the incidence of tracheal intubation was not statistically significant (OR = 0.74, 95% CI (0.34–1.59), *Z* = 0.78, *P* = 0.44, Fig. 4C).

#### The length of hospital stay

**Fig. 5:**
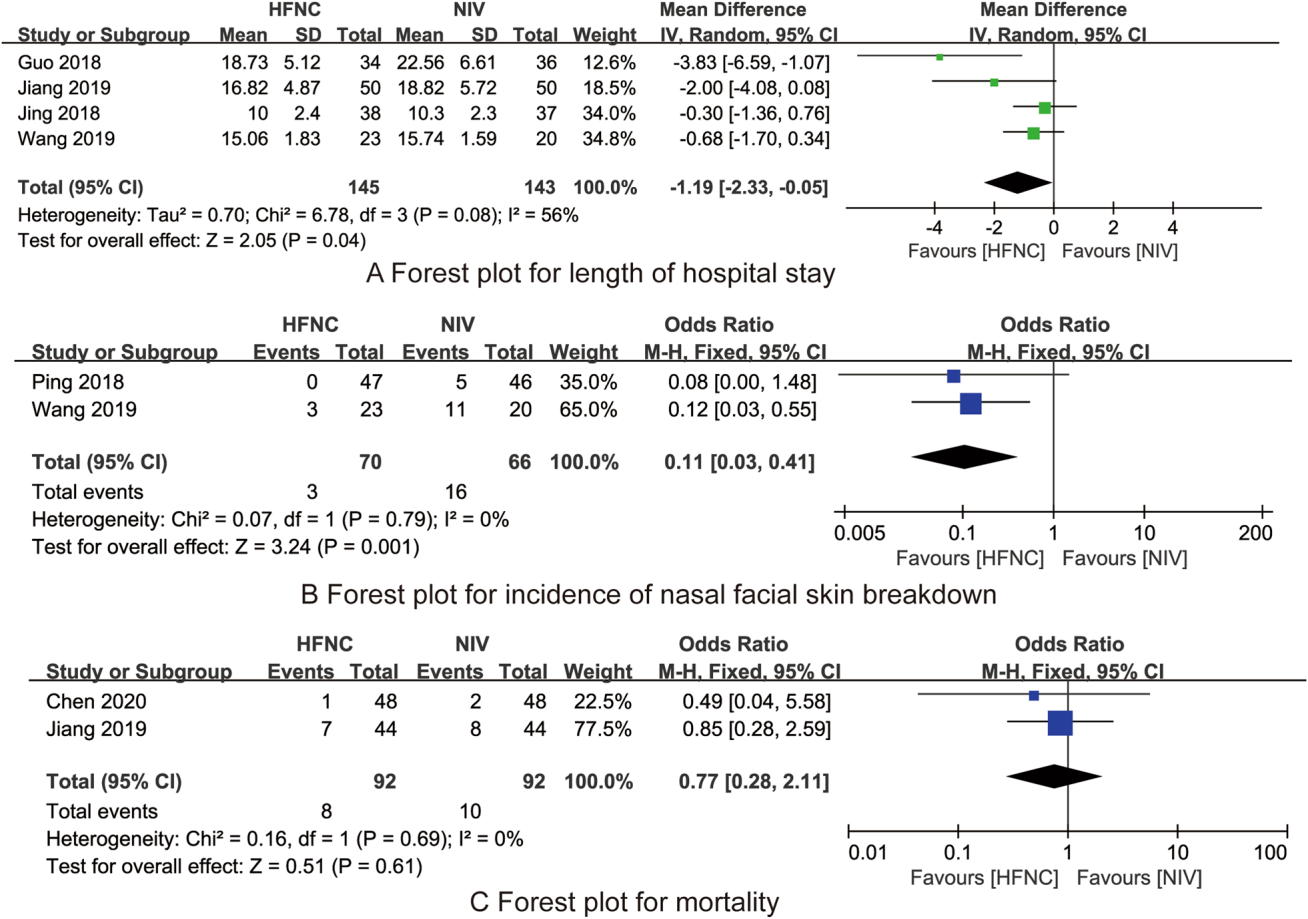
The forest plots for synthesized outcomes

Four RCTs [19, 20, 22, 24] compared the effects of HFNC and NIV on the length of hospital stay. There was heterogeneity between the studies (*I*^2^ = 56%, *P* = 0.08), and the random effects model was used for analysis. The results showed that compared with NIV, HFNC could significantly reduce the length of hospital stay (MD = − 1.19, 95% CI (− 2.23 to − 0.05), *Z* = 2.05, *P* = 0.04, Fig. 5A).

#### The incidence of nasal facial skin breakdown

Two RCTs [19, 21] compared the effects of HFNC and NIV on the incidence of nasal facial skin breakdown. There was no heterogeneity between the studies (*I*^2^ = 0%, *P* = 0.79), and the fixed effects model was used for analysis. The results showed that compared with NIV, HFNC could significantly reduce the incidence of nasal facial skin breakdown (OR = 0.11, 95% CI (0.03–0.41), *Z* = 0.51, *P* = 0.61, Fig. 5B).

#### Mortality

Two RCTs [23, 24] compared the effects of HFNC and NIV on the mortality. There was no heterogeneity between the studies (*I*^2^ = 0%, *P* = 0.69), and the fixed effects model was used for analysis. The results showed that the difference between the two groups in the mortality was not statistically significant (OR = 0.77, 95% CI (0.28–2.11), *Z* = 0.51, *P* = 0.61, Fig. 5C).

## Discussion

The main reason for the hospitalization of COPD patients is due to the continuous progress of the disease and severe respiratory failure [18]. Emergency tracheal intubation is often used during the treatment process, and invasive ventilator-assisted ventilation is required [25]. HFNC oxygen therapy is a new type of treatment that can provide patients with stable oxygen concentration, improve mucosal clearance, prevent nasopharyngeal dead space and open alveoli, so it has great clinical promotion value [26]. The main factors of organ dysfunction in patients with type II respiratory failure are respiratory acidosis, hypercapnia and hypoxemia [27]. When the PaCO_2_ level in the body rises to a certain level, the PaO_2_ level usually decreases, leading to cardiac arrest, pulmonary heart disease, etc., which will have a serious impact on the life of the patient [28, 29]. However, PaCO_2_ is used to determine the ventilation status of the alveoli, and its increase indicates that the lungs are insufficiently ventilated. PaO_2_ is a sensitive indicator of hypoxia in the body. Decreased PaO_2_ is seen in ventilation dysfunction. PaCO_2_ and PaO_2_ are not necessarily proportional. Once hyperventilation sets in, blood gas exchange in the patient’s alveoli is restricted; PaO_2_ may also decrease significantly [30]. The results of our meta-analysis have found that compared with NIV, HFNC is more beneficial in reducing the PaCO_2_ level, length of hospital stay and the incidence of nasal facial skin breakdown, no effect differences in the PaO_2_, the incidence of tracheal intubation and mortality were found between HFNC and NIV treatments. A similar study published by Yang et al. [31] has compared HFNC and conventional NIV to evaluate the mortality and intubation risk in AECOPD in 8 RCTs and 492 patients, and it had resulted a low-quality evidence for HFNC not increasing mortality and intubation risk. Another meta-analysis published by Huang et al. [32] has showed no differences on PaO2 levels between patients treated with HFNC vs NIV, but with a smaller study population. In our study, HFNC has showed superiority compared to NIV in hospital LOS and PaCO_2_ levels, which are different from previous two meta-analyses. The reasons may be that a bias could have occurred in selecting patients, for example including also mild patients, and the included RCTs are from different populations and areas.

HFNC can significantly improve CO_2_ retention in COPD patients, and its influence on PaO_2_ levels and tracheal intubation rate needs further study. The results of a multicenter, randomized controlled crossover trial [33] showed that HFNC reduced PaCO_2_ levels no less than NIV. At the same time, multiple studies [34, 35] have shown that compared with the long-term oxygen therapy group, HFNC can significantly reduce PaCO_2_ levels and improve hypercapnia. This may be because HFNC can provide high-flow gas, which can meet or exceed the peak inspiratory flow of patients with dyspnea, thus avoiding the dilution of the inhaled gas due to inhaling too much indoor air [36]. At the same time, it provides high-velocity air flow. By flushing the upper respiratory tract, the mixed gas reduces the ineffective lung anatomy, which not only allows the patient to expel CO_2_ from the anatomical ineffective cavity during exhalation, but also greatly reduces the repeated inhalation of CO_2_, thereby effectively reducing the PaCO_2_ level [37, 38]. Theoretically, HFNC can generate a positive end-expiratory pressure of 2–5 cmH_2_O to provide sufficient oxygen to enter the alveoli and improve oxygenation [39]. However, compared with NIV, there is no significant difference in efficacy. This may be because although HFNC can produce a certain amount of positive end-expiratory pressure, it does not have the ventilatory support function of NIV [40]. Some patients with weak respiratory muscles may benefit from the ventilatory support function of NIV [41].

HFNC can reduce the occurrence of facial pressure injuries and improve the comfort of COPD patients [42]. HFNC can provide patients with gas heated and humidified up to 37 °C, and the temperature of the gas entering the human body is similar to normal body temperature [43]. HFNC delivers the heated and humidified gas directly to the nasal cavity, which makes up for the insufficient heating and humidification effect of the heating and humidifying gas provided by the NIV when it passes through the pipe or mask because it is not continuously heated and cooled, thereby reducing airway mucosal damage [44]. Moreover, HFNC provides continuous high-flow oxygen through the nasal cannula. Compared with NIV, it can significantly reduce the incidence of pressure injuries such as facial and nasal bridge rupture [45]. HFNC makes up for the insufficient heating and humidification of traditional oxygen therapy, and at the same time avoids the problems of oropharyngeal dryness, intolerance, and facial pressure injury caused by inhalation of pressurized gas in NIV treatment [46]. It is also convenient for patients to cough, eat and talk [47]. Therefore, HFNC can effectively improve the comfort of patients.

Studies [48, 49] have shown that HFNC treatment can effectively improve the blood gas index of children. Besides, HFNC treatment can effectively improve the heart rate and respiratory rate of patients with acute respiratory failure after extubation of surgically invasive mechanical ventilation. HFNC can continuously provide patients with high-flow oxygen with constant oxygen concentration, and generate positive airway pressure, which can increase the end-tidal volume, help CO_2_ discharge, reduce physiological dead space, and effectively improve clinical symptoms, thereby improving the results of blood gas analysis [50]. Meanwhile, it can increase the oxygenation index, reduce the average arterial pressure, heart rate and respiratory rate [51].

This study has certain limitations that must be considered. Firstly, the included RCTs are all from China, and the quality of the literature is average, and there may be certain regional and population differences. Therefore, we must be cautious about the authenticity and extrapolation of the results of this study. Secondly, due to the small number of included RCTs and small sample size, the statistical power of the research may be not enough to detect the differences. Thirdly, in this particular period, during COVID-19 epidemic phase, the role of HFNC, which carries large amounts of oxygen, in the correction of CO_2_ retention is less clear. In the future, multi-center large-sample studies are needed to confirm the exact efficacy and safety of HFNC for COPD patients, and to clarify the specific beneficiaries of COPD patients using HFNC.

## Conclusions

In summary, compared with NIV, HFNC can reduce the PaCO_2_ level, length of hospital stay and the incidence of nasal facial skin breakdown, and there are not effect differences in the PaO_2_, the incidence of tracheal intubation and mortality between HFNC and NIV treatments. We recommend HFNC therapy use based on our results, yet currently NIV is more used in some poor or underdeveloped areas with less expenditure and convenience; more studies on the effects and costs of NIV and HFNC therapy are needed. This meta-analysis is limited by sample size and quality of included RCTs; more high-quality studies with large sample (better ≥ 166 cases based on power calculation) in different areas are needed to further elucidate the role of HFNC in the treatment of COPD and respiratory failure.

## Data Availability

All data generated or analyzed during this study are included in this published article.

## Abbreviations

COPD: Chronic obstructive pulmonary disease
NIV: Noninvasive ventilation
HFNC: High-flow nasal cannula
OR: Odds ratio
95% CI: 95% Confidence interval
MD: Mean difference
